# Computed tomography-based radiomics and body composition model for predicting hepatic decompensation

**DOI:** 10.18632/oncotarget.28673

**Published:** 2024-11-22

**Authors:** Yashbir Singh, John E. Eaton, Sudhakar K. Venkatesh, Bradley J. Erickson

**Affiliations:** ^1^Department of Radiology, Mayo Clinic, Rochester, MN 55905, USA; ^2^Division of Gastroenterology and Hepatology, Mayo Clinic, Rochester, MN 55905, USA

**Keywords:** radiomics, body composition, machine learning, primary sclerosing cholangitis, computer tomography

## Abstract

Primary sclerosing cholangitis (PSC) is a chronic liver disease characterized by inflammation and scarring of the bile ducts, which can lead to cirrhosis and hepatic decompensation. The study aimed to explore the potential value of computational radiomics, a field that extracts quantitative features from medical images, in predicting whether or not PSC patients had hepatic decompensation. We used an in-house developed deep learning model called the body composition model, which quantifies body composition from computed tomography (CT) into four compartments: subcutaneous adipose tissue (SAT), skeletal muscle (SKM), visceral adipose tissue (VAT), and intermuscular adipose tissue (IMAT). We extracted radiomics features from all four body composition compartments and used them to build a predictive model in the training cohort. The predictive model demonstrated good performance in validation cohorts for predicting hepatic decompensation, with an accuracy score of 0.97, a precision score of 1.0, and an area under the curve (AUC) score of 0.97. Computational radiomics using CT images shows promise in predicting hepatic decompensation in primary sclerosing cholangitis patients. Our model achieved high accuracy, but predicting future events remains challenging. Further research is needed to validate clinical utility and limitations.

## INTRODUCTION

Primary Sclerosing Cholangitis (PSC) is a chronic cholestatic liver disorder characterized by inflammation and fibrosis of the extra and/or intrahepatic bile ducts, which can lead to cirrhosis and complications stemming from portal hypertension [1, 2]. Hepatic decompensation refers to the development of serious complications in patients with advanced liver disease, including ascites, variceal bleeding, hepatic encephalopathy, or jaundice. Early detection of hepatic decompensation plays a vital role in facilitating prompt therapeutic interventions, which can enhance clinical outcomes and optimize treatment strategies. Biomarkers to predict clinically significant events are important, as this allows for the risk stratification of patients in clinical practice and in clinical trials. However, there is not yet a reliable way for early identification of which patients with PSC will experience hepatic decompensation. Body composition has been used to predict outcomes in other chronic diseases, such as cancer and cardiovascular disease [3, 4]. Our research implements an in-house-developed deep learning approach for quantitative body composition analysis. This model performs comprehensive tissue segmentation, specifically identifying and measuring subcutaneous adipose tissue (SAT), skeletal muscle (SKM), visceral adipose tissue (VAT), and intermuscular adipose tissue (IMAT). The segmentation framework employs a convolutional neural network utilizing the U-Net architecture, which was trained using a substantial dataset comprising 2,430 two-dimensional Computed Tomography (CT) abdominal scans. Body composition analysis quantifies the distribution patterns of muscular and adipose tissues throughout the body, providing valuable insights into various pathological conditions including cancer and cardiovascular disorders that correlate with patient outcomes [3–5].

The low density (Hounsfield unit) range of adipose tissue makes it easier to distinguish from other tissues. The subcutaneous adipose tissue and bone compartment had the highest Dice and Jaccard scores, while the visceral adipose tissue had the lowest. The visceral adipose tissue compartment had more variability than the other compartments.

Radiomics include multiple classes of quantitative imaging characteristics that each captures a different property in a region of interest (ROI). First-order, for example, intensity, and second-order statistical features, such as texture, contrast, and homogeneity, are the most common radiological features. The former is calculated using the histogram of grey-level pixels, irrespective of spatial relationships among the pixels. The ROI shapes and edge detection capabilities underlying object boundaries include morphological functions in other radiomic features [6–9]. We developed a method using Computed Tomography-Based Radiomics Signature and the Body Composition Model to predict hepatic decompensation, given this promising technique and the unmet need to better predict unfavorable outcomes in those with PSC.

## RESULTS

In predicting hepatic decompensation for PSC patients, the computational model achieved favorable performance metrics. The model utilized a combination of CT-based radiomics signature and a deep learning-based body composition model. It was trained on a dataset of 80 patients, including 30 with hepatic decompensation, 30 without decompensation, and 20 in an external validation set. The model’s performance was evaluated using the area under the receiver operating characteristic curve (AUC), a metric that quantifies the prediction performance of a binary classifier. Notably, the model achieved an impressive AUC of 0.97 on the validation set, indicating its exceptional ability to discriminate between patients who would experience hepatic decompensation and those who would not. Furthermore, the model demonstrated a prediction accuracy of 97% on the validation set, which represents the percentage of correct classifications made by the model (Table 1). This high accuracy highlights the model’s potential for accurately identifying patients at risk of hepatic decompensation, enabling timely clinical interventions and personalized treatment strategies.

**Table 1:** Predicted values for metrics obtained during a 5-fold stratified cross-validation evaluation of the random forest classifier in the validation cohort

Metric	Fold 1	Fold 2	Fold 3	Fold 4	Fold 5
Accuracy score	0.94	0.94	0.87	1.0	0.98
Precision-score	0.89	0.94	0.83	1.0	1.0
Recall Score	1.0	0.93	0.93	1.0	0.94
AUC score	0.94	0.94	0.87	1.0	0.97

## DISCUSSION

We have developed a novel method that predicts hepatic decompensation in patients with PSC by combining CT-Based Radiomics Signature and a Body Composition Model. This approach leverages the power of advanced imaging analysis techniques to extract valuable information from a vast amount of imaging data, enabling accurate classification of hepatic decompensation in PSC patients.

Our study represents a pioneering proof-of-concept application that integrates radiomics signatures and body composition analysis from CT scans. The proposed model was designed to predict short-term outcomes, serving as a crucial first step in this innovative approach. However, further research is necessary to validate our findings on a large-scale, independent dataset, ensuring the robustness and generalizability of the model.

The potential applications of this methodological approach extend beyond the current scope. It may hold promise for the detection of other PSC-related complications, such as cholangiocarcinoma, as well as applications in more prevalent chronic liver diseases like non-alcoholic fatty liver disease (NAFLD) [10, 11]. By harnessing the wealth of information contained within imaging data, our approach could pave the way for improved risk stratification, personalized treatment strategies, and ultimately, better patient outcomes.

It is crucial to note that this study represents an initial step. Given its small sample size and single-center design, further investigations are needed to refine and validate the proposed model. Nonetheless, the integration of radiomics signatures and body composition analysis presents an exciting avenue for advancing our understanding and management of PSC and other chronic liver diseases.

We have demonstrated a set of imaging biomarkers extracted from a body composition model with PyRadiomics from CT scans. This study reveals the potential for prognostic features in predicting hepatic decompensation in patients with PSC. It provides hidden information that will aid in the discovery of new differentiating imaging features, paving the way for improved risk stratification and personalized treatment strategies for PSC and other chronic liver diseases.

## MATERIALS AND METHODS

Our study protocol received approval from Mayo Clinic’s Institutional Review Board (IRB) (18-009852) in Rochester, MN, and adhered to the 1975 Declaration of Helsinki’s ethical standards, with informed consent obtained from all participants. Study enrollment required both a confirmed PSC diagnosis and portal venous phase abdominal CT imaging. The imaging protocol standardized contrast administration (2–3 ml/sec, weight-based) with consistent portal venous phase timing (70-second delay). The final cohort included 80 subjects distributed across three groups: 30 patients with previous hepatic decompensation, 30 without decompensation history, and 20 patients allocated for external validation within a 5-fold cross-validation framework. This was a retrospective cohort study with CT studies obtained from 1993 to 2021. The study excluded patients with prior or concurrent hepatic decompensation events, diagnosed cholangiocarcinoma, or previous liver transplantation relative to their CT examination date. Following the baseline CT scan, patients underwent monitoring until they experienced hepatic decompensation, underwent liver transplantation, or reached their last follow-up appointment. The surveillance duration had a median of 1.5 years (range: 142–1, 318 days).

### Body composition model

**Figure 1:**
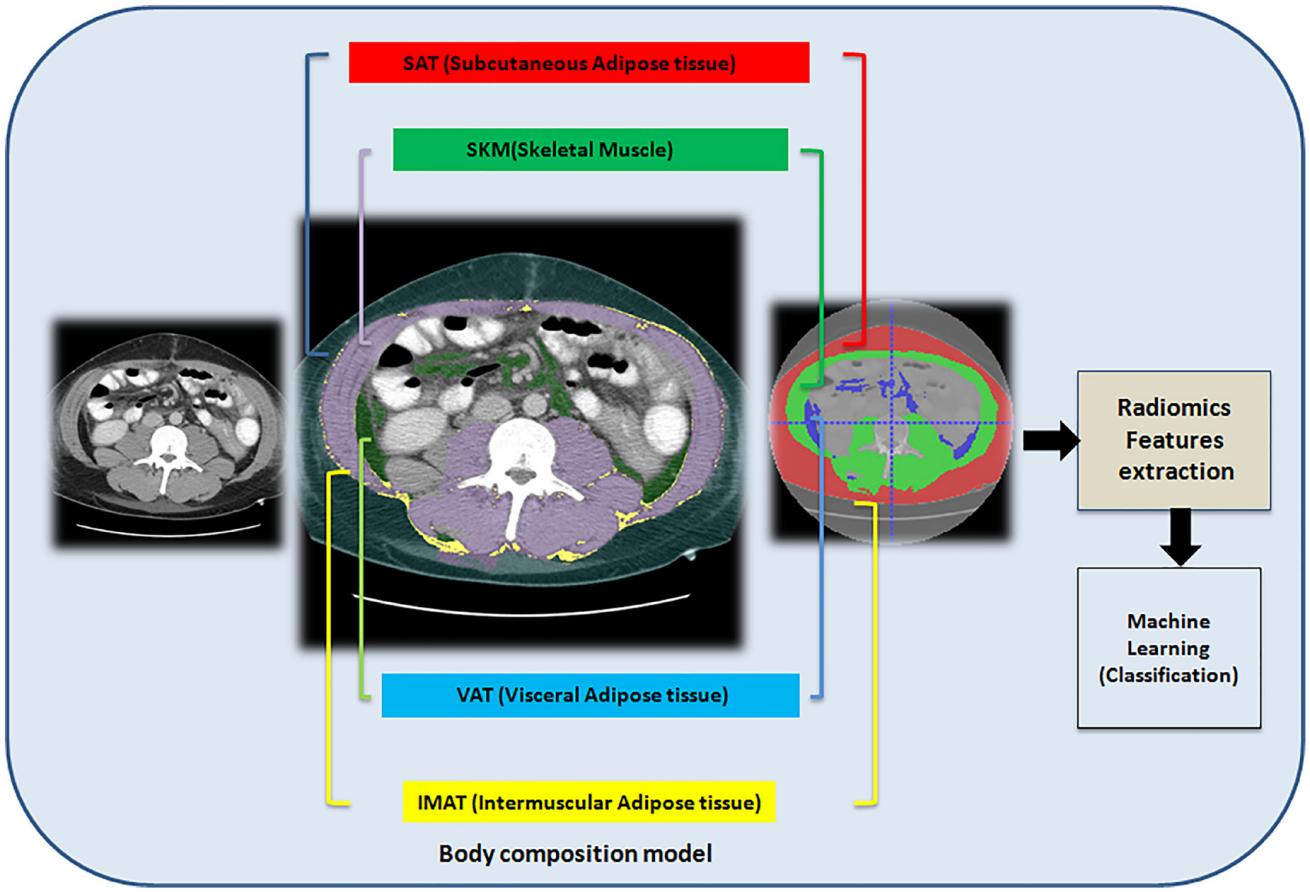
Workflow of the prediction of primary sclerosis cholangitis using computed tomography-based radiomics signature and the body composition model.

This is a U-Net model developed in-house that assesses body composition using the metrics SAT, SKM, VAT, and IMAT (Figure 1). The idea of “body composition” describes how the distribution of muscle and fat in the body varies in situations like cancer and chronic diseases, which are associated with clinical outcomes [4, 5]. This advanced model utilizes deep learning techniques to accurately quantify these tissue compartments from CT images, providing a comprehensive analysis of an individual’s body composition. By offering detailed insights into the proportions and distributions of these key tissue types, the model enables clinicians to better understand a patient’s metabolic health, assess disease risk, and potentially predict treatment outcomes. This non-invasive approach to body composition analysis represents a significant advancement in personalized medicine and risk stratification.

### Feature extraction

We used the PyRadiomics library, an open-source Python package, to extract radiomic features on the ROI which is created by the body composition model. We included descriptors for the ROI’s two-dimensional size and shape. Because these features are independent of the ROI’s gray level intensity distribution, they can only be calculated on the non-derived image and mask. This class can only be calculated for truly 2D masks. Force2Ddimension must be set to the dimension that is out of plane (e.g., 0 (z-axis) for an axial slice) to ensure proper processing. Statistical analysis utilizing *t*-test methodology revealed 23 significant radiomics features from a comprehensive set of 100 features, as referenced in the PyRadiomics documentation portal (https://pyradiomics.readthedocs.io/en/latest/features.html) [9].

### Machine learning model

We aimed at developing a classification model using radiomics features based upon a traditional machine learning approach (random forest classification). The random forest produces several decision trees by selecting random subsets of features to classify all smaller trees according to the mode (for classification) or the average (for regression). The extracted features served as input for our classifier. We used scikit-learn (version 0.24.2) to train class sklearn.ensemble: RandomForestClassifier and used default parameters except for max leaf nodes.

## Abbreviations

PSC: Primary sclerosing cholangitis
CT: computed tomography
AUC: area under the curve
SAT: subcutaneous adipose tissue
SKM: skeletal muscle
VAT: visceral adipose tissue
IMAT: intermuscular adipose tissue
ROI: region of interest
